# Dataset demonstrating the temperature effect on average output polarization for QCA based reversible logic gates

**DOI:** 10.1016/j.dib.2017.06.058

**Published:** 2017-07-04

**Authors:** Md. Kamrul Hassan, Nur Mohammad Nahid, Ali Newaz Bahar, Mohammad Maksudur Rahman Bhuiyan, Md. Abdullah-Al-Shafi, Kawsar Ahmed

**Affiliations:** a Department of Information and Communication Technology, Mawlana Bhashani Science and Technology University, Bangladesh; b University Grants Commission of Bangladesh, Bangladesh; c Institute of Information Technology (IIT), University of Dhaka, Bangladesh

## Abstract

Quantum-dot cellular automata (QCA) is a developing nanotechnology, which seems to be a good candidate to replace the conventional complementary metal-oxide-semiconductor (CMOS) technology. In this article, we present the dataset of average output polarization (AOP) for basic reversible logic gates presented in Ali Newaz et al. (2016) [1]. QCADesigner 2.0.3 has been employed to analysis the AOP of reversible gates at different temperature levels in Kelvin (K) unit.

**Specifications Table**

**Table:** 

Subject area	*Electronics*
More specific subject area	*Nano-electronics*
Type of data	*Table, figure*
How data was acquired	*Data set has been acquired using QCADesigner tool*
Data format	*Analyzed*
Data accessibility	*Data is within this article*

**Value of the data**

• Reversible gates are the basic building block of reversible logic systems. This dataset helps researcher to enhance the performance and reliability of digital systems.
• The presented data analysis can support the researchers to find the maximum operating temperature of a particular QCA design.
• The proposed dataset can be used to design robust and lossless arithmetic logic unit (ALU) in quantum computers.

## 1 Data

This article describes the average output polarization (AOP) for basic reversible logic gates of Double Feynman, Toffoli, TR, R, NG, SCL and BVF gates at different temperature levels are shown in Table 1.

**Table 1:** Average output polarization (AOP) dataset of reversible logic gates at different temperature levels.

Reversible Gate	Output cell	Average output polarization (AOP)											
		Temperature											
		1	2	3	4	5	6	7	8	9	10	11	12
Double Feynman Gate	*P*	3.518	3.504	3.500	3.500	3.493	3.485	3.466	3.448	3.384	3.308	3.239	3.155
	*Q*	3.511	3.509	3.507	3.506	3.500	3.493	3.471	3.434	3.386	3.324	3.247	3.166
	*R*	3.505	3.505	3.505	3.503	3.500	3.489	3.465	3.430	3.381	3.315	3.237	3.142
Toffoli Gate	*P*	3.515	3.505	3.503	3.501	3.500	3.495	3.473	3.440	3.396	3.337	3.267	3.190
	*Q*	3.510	3.507	3.503	3.500	3.500	3.481	3.469	3.449	3.378	3.330	3.257	3.180
	*R*	3.508	3.506	3.506	3.504	3.500	3.489	3.465	3.430	3.381	3.315	3.241	3.155
TR Gate	*P*	3.515	3.510	3.506	3.503	3.501	3.491	3.469	3.436	3.383	3.322	3.248	
	*Q*	3.509	3.508	3.506	3.502	3.500	3.487	3.465	3.429	3.375	3.315	3.241	
	*R*	3.502	3.502	3.502	3.501	3.499	3.482	3.460	3.425	3.369	3.308	3.231	
R Gate	*P*	3.506	3.505	3.503	3.503	3.490	3.487	3.460	3.420	3.370	3.310		
	*Q*	3.518	3.515	3.510	3.505	3.501	3.500	3.491	3.457	3.399	3.360		
	*R*	3.502	3.502	3.501	3.500	3.499	3.487	3.466	3.427	3.375	2.107		
NG Gate	*P*	3.515	3.513	3.513	3.513	3.510	3.499	3.480	3.447	3.394	3.335	3.265	3.186
	*Q*	3.502	3.502	3.501	3.501	3.495	3.481	3.458	3.422	3.371	3.309	3.232	3.140
	*R*	3.509	3.509	3.509	3.505	3.500	3.489	3.465	3.430	3.381	3.315	3.241	3.159
SCL Gate	*P*	3.518	3.515	3.510	3.507	3.506	3.499	3.490	3.540	3.396	3.346	3.280	3.199
	*Q*	3.513	3.510	3.507	3.502	3.501	3.497	3.483	3.500	3.389	3.338	3.280	3.180
	*R*	3.510	3.509	3.505	3.501	3.500	3.490	3.473	3.440	3.380	3.330	3.258	3.160
	*S*	3.506	3.506	3.506	3.504	3.500	3.489	3.465	3.430	3.380	3.315	3.241	3.148
BVF Gate	*P*	3.513	3.510	3.508	3.504	3.500	3.489	3.479	3.435	3.399	3.320	3.240	3.169
	*Q*	3.518	3.515	3.513	3.510	3.505	3.500	3.495	3.478	3.420	3.380	3.270	3.199
	*R*	3.509	3.507	3.502	3.501	3.500	3.490	3.470	3.437	3.390	3.347	3.264	3.180
	*S*	3.504	3.504	3.504	3.504	3.499	3.487	3.466	3.427	3.379	3.315	3.237	3.149

## 2 Experimental design, materials and methods

### 2.1 AOP analysis

The average output polarization is declined gradually with the increment of temperature [2]. At any specific temperature, the AOP of an output cell can be calculated by simply taking the difference between maximum polarization and minimum polarization and dividing the result by two.

(1) $$\mathrm{AOP}=\frac{\mathrm{maximum}-\mathrm{minimun}}{2}$$

**Fig. 1:**
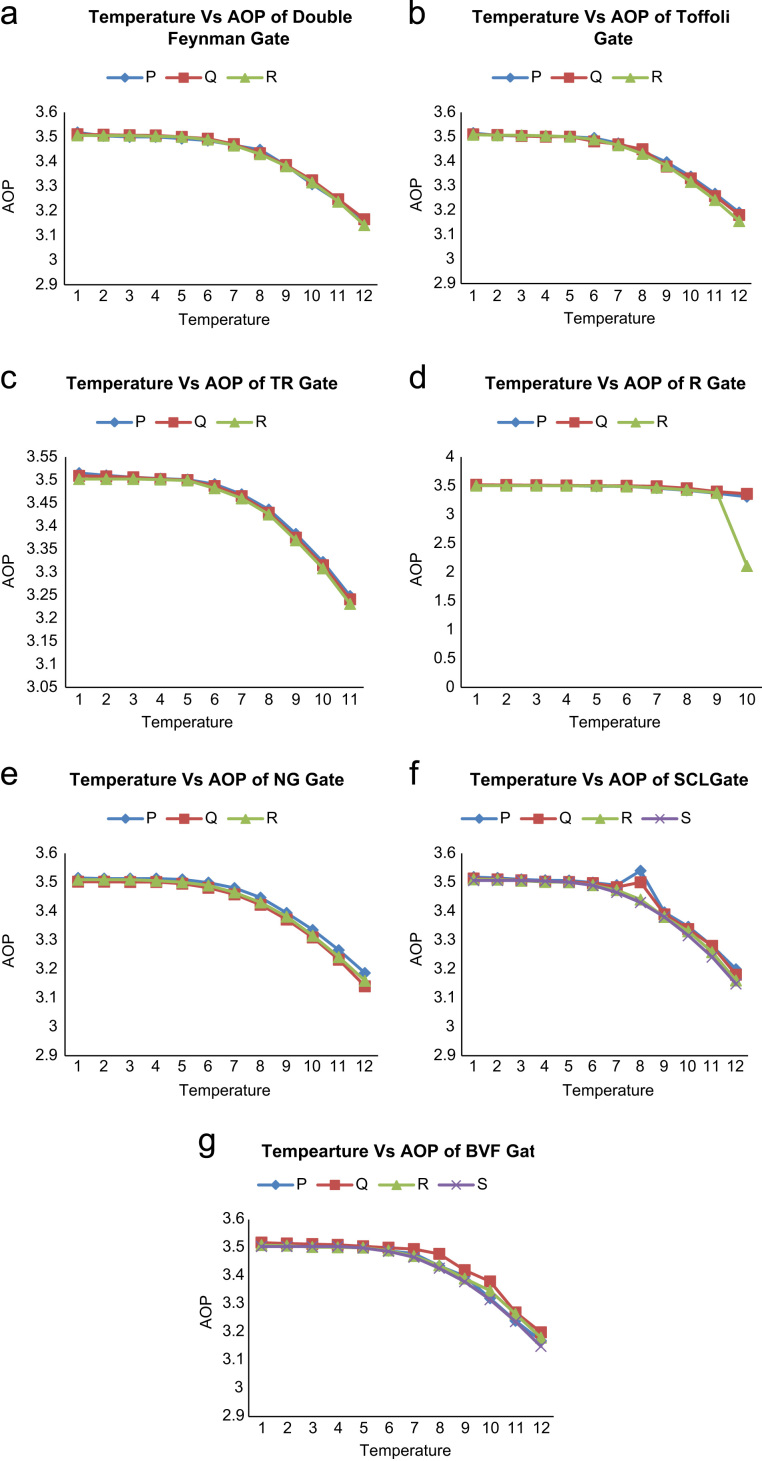
Temperature effect on average output polarization (AOP) of (a) Double Feynman gate (b) Toffoli gate (c) TR gate (d) R gate (e) NG gate (f) SCL gate (g) BVF gate.

To analysis the AOP, QCADesigner tool ver. 2.0.3 [3] has been used with coherent vector simulation engine. The following default parameters have been considered. The default parameters are listed as: QCA cell size = 18 nm, diameter of quantum dots = 5 nm, number of samples = 50,000, relative permittivity = 12.9, convergence tolerance = 0.001, radius of effect = 65 nm, clock low = 3.8e^−23^ J, clock high = 9.8e^−22^ J, clock amplitude factor = 2.000, layer separation = 11.5 nm and maximum iterations per sample = 100. The graphical representation of AOP of different reversible logic gates presented in [1] is illustrated in Fig. 1.
